# Dental Origin of Pyæmia

**Published:** 1888-07

**Authors:** 


### Editorial, Etc.

---

Dental Origin of Pyæmia.—Dr. Arthur Baker in a paper read before the Royal Academy of Medicine in Ireland and published in the *Dublin Journal of Medical Science*, says:

As pyæmia from dental causes is not very common, or perhaps has not been frequently recognized, the notes of a case which came under my own observation are, I think, deserving of record. Before, however, detailing my own case, I shall refer to some of the scanty literature on the subject.

In the recent edition of Tomes' "Dental Surgery," the author refers to seven cases of acute and chronic pyæmia of dental origin which he has collected from various sources, all of which proved fatal. Two of these, however, in my opinion were, strictly speaking, not pyæmic, as we now understand the term. In one, extension of an abscess connected with a lower wisdom tooth produced some severe unilateral glossitis, and mechanically suffocated the patient. In the other, splintering of the lower jaw in the removal of a tooth gave rise to an abscess, which found its way up the ramus, and through the foramina, ovale, rotundum, and spinosum, into the cavity of the cranium, where meningitis ensued, of which the patient died. The remaining five cases may fairly be classed as pyæmic, and as such deserve our attention, for they point to the fact that alveolar suppuration cannot always be treated as an affection of no moment, but may sometimes be attended with even fatal consequences. Unfortunately, details are wanting in the cases recorded by Mr. Tomes, upon which we might with advantage generalize.

Dr. R. J. Porre, at the International Medical Congress held at Washington in 1887, brought forward some cases of chronic pyæmia of dental origin, in all of which there was, fortunately, a happier termination than in those recorded by Mr. Tomes. Dr. Porre gives the following account of one of the cases observed by him :—The patient, male, good constitution and habits, suffered for the last thirty years from neuralgia, besides having constantly recurring furuncles and eruptions in various parts of the body, which would often for months become running abscesses. He experienced burning and itching eruptions of hands and feet, whlch would finally change to stubborn ulcerations. His bowels were either stubbornly constipated or exhaustingly loose. He suffered from frequent rigors and febrile attacks of varying intensity, profuse night sweats, retention of urine, serious constrictions of the bowels and urethra. Lancinating pains darted from the maxilla of right side to bowels, bladder, limbs, hands and feet, or to whatever part was locally affected at the time. This latter peculiarity, together with the discovery of a little pus exuding from the locality of the wisdom tooth, led to a final correct diagnosis of his case. The tooth referred to was extracted, and a speedy and complete recovery followed. Dr. Porre also read the notes of ten similar cases, which all yielded to the simple remedy of removing the offending tooth.

Mr. Frederick Eve, Curator of the Royal College of Surgeons, England, in a recent communication to the Odontological Society, whilst noticing the fact that in periodontal abscess, although the pus directly gained access to the bone, serious consequences rarely followed—yet related the case of a young man who had been troubled by an abscess in the region of the fangs of a second molar tooth. He attended the funeral of his grandfather, caught cold in the tooth, and died in three weeks of pyæmia.

Some interesting experiments on mice are at present being carried out by Dr. W. D. Miller of berlin, by inoculating them with the material obtained from gangrenous pulps. As these experiments are not yet concluded, it would be premature to do more than allude to them here; but from Dr. Miller's recent communication to the *Dental Cosmos*, he is evidently quite alive to the importance of investigating this source of pyæmia.

The following case occured in my own practice. I am happy to say that it was not fatal, and that I succeeded in curing the patient without sacrificing the offending tooth.

CASE.—Mrs.——, aged thirty-three, widow, consulted me in the beginning of November, 1887, about her first left upper molar tooth, which had given her pain from time to time. The patient appeared to be in excellent health, and presented no evidence of constitutional taint. She stated that the only illness she had had, excepting, of course, her confinements was a severe attack of scarlatina at twelve years of age which was followed by general dropsy. At the age of nineteen, the first left upper molar (the tooth about which she consulted me) was filled; this resulted in an alveolar abscess over the tooth. Ten years later she had another rather acute abscess in the same place, which was followed at once by a small abscess on the fourth toe of the right side; then small abscesses broke out over other parts of the body at the same time, the tooth being the seat of more or less uneasiness. Early in the spring of 1887, she suffered from an abscess in the right ear, which by the medical attendant was thought to be connected with some tooth. About a week previous to consulting me, she had a recurrence of the abscess over the left upper molar; this was followed by a small pimple on the back of the right forearm, which at the time of her visit presented the appearances of a small pyæmic abscess. Viewing the case as one of chronic pyæmia, having its origin in the suppuration about the roots of the molar, I decided to try and save the tooth, and in doing so, if possible, to cure the pyæmia.

The tooth I treated by removing all that remained of the dead and decomposed pulp, cleaning out the roots thoroughly syringing them first with weak carbolic lotion, then with absolute alcohol. The roots were finally dried with hot air and injected with a solution of iodoform in ether, and the filling of the tooth was completed at a subsequent sitting. There was a swelling, about the size of a small marble, on the gum over the buccal roots of the tooth, corresponding to the site of the alveolar abscess to which I have alluded; this I laid open freely, and allowed it to heal from the bottom. The fluid which escaped from this swelling was more like cystic fluid, such as is frequently found as the result of chronic inflammation round the roots of teeth, than true pus.

It is now more than six months since I treated this case, and being interested as to the result, I have kept the patient under observation. She has no further trouble with the tooth, and the metastatic abscesses have ceased. That the tooth was the source of the poison, to my mind appears extremely probable, both from the frequency with which the periodontal abscess was succeeded by an abscess elsewhere, and the fact that the small abscess, which was situated on the patient's fore arm when she came to me, subsided more rapidly than any previous abscess on treatment of the tooth; and no abscess has since then appeared.

I am not quite clear as to the explanation of why the pyæmia evidence itselt always on the right side, while its dental origin was on the left. That more serious symptoms did not present themselves in this case was most likely due, as pointed out by Mr Watson Cheyne in his recent lectures on "Suppuration and Septic Diseases," to the small dose of the poison.

